# Extracorporeal Free-Light-Chain Removal in Myeloma Cast Nephropathy: Plasma Exchange and Hemodialysis Strategies in the Era of Effective Antimyeloma Therapy

**DOI:** 10.3390/jcm15187211

**Published:** 2026-09-17

**Authors:** Guido Gembillo, Luca Soraci, Lorenzo Lo Cicero, Chiara Terzo, Chiara Casuscelli, Felicia Cuzzola, Flavia Sudano, Maria Federica Ricca, Manuel Scapellato, Rossella Messina, Alberto La Spada, Ankit Sharma, Domenico Santoro

**Affiliations:** 1 Unit of Nephrology and Dialysis, Department of Clinical and Experimental Medicine, University of Messina, 98125 Messina, Italy; lorenzolocicero95@gmail.com (L.L.C.);; 2 Unit of Geriatric Medicine, Italian National Research Center on Aging (IRCCS INRCA), 87100 Cosenza, Italy; l.soraci@inrca.it; 3 UOSD Nephrology and Dialysis, Hospital “G. Di Maria”, Contrada Chiusa di Carlo, 96012 Avola, Italy; 4 Renal Department, University Hospitals of Coventry and Warwickshire, Coventry CV2 2DX, UK; ankit.sharma1@nhs.net

**Keywords:** multiple myeloma, cast nephropathy, acute kidney injury, hemodialysis, free light chains, high-cutoff membranes, medium-cutoff membranes, extracorporeal removal

## Abstract

Renal impairment affects up to half of patients with newly diagnosed multiple myeloma, and roughly 2–4% require dialysis at presentation, most often because of light-chain cast nephropathy. In these patients, renal recovery is associated with the speed and magnitude of serum free light-chain (sFLC) reduction in observational cohorts, an association that mainly reflects the hematologic response to chemotherapy; the incremental benefit of removing circulating sFLCs by extracorporeal means remains uncertain, and this narrative review is organized around that question in the current era of effective antimyeloma therapy. Plasma exchange was tested in three small randomized trials before proteasome inhibitors were available, the largest finding no benefit, and two randomized trials of high-cutoff hemodialysis on a bortezomib backbone, MYRE and EuLITE, found no benefit for their primary renal endpoints, with more pulmonary infections in the high-cutoff arm of EuLITE. Medium-cutoff membranes, polymethylmethacrylate adsorptive dialyzers, and hemodiafiltration with endogenous reinfusion have achieved substantial sFLC reduction with less albumin loss in small uncontrolled series, without any randomized comparison against conventional dialysis. Daratumumab-containing induction lowers sFLCs within days and may further narrow the residual rationale for enhanced clearance. Conventional high-flux hemodialysis combined with prompt antimyeloma therapy is therefore the evidence-based standard kidney-replacement modality when dialysis is indicated; the patient subsets in which a time-limited enhanced-removal strategy might still be tested are proposed as hypotheses for prospective trials, without validated thresholds.

## 1. Introduction

Multiple myeloma (MM) accounts for approximately 1% of all malignancies and 10% of hematologic malignancies, with a worldwide age-standardized incidence of nearly 1.8 per 100,000 and a median age at diagnosis typically in the seventh decade [1,2]. Renal involvement is a conspicuous feature from the outset; up to half of patients present with an estimated glomerular filtration rate (eGFR) below 60 mL/min/1.73 m^2^. Severe acute kidney injury (AKI), variably defined across these series, is considerably less common, and 2–4% are already dialysis-dependent at the time of myeloma identification [3,4,5]. This latter subgroup represents a prognostically distinct cohort. Prior to the availability of proteasome inhibitors, a substantial proportion of patients with dialysis-dependent AKI secondary to cast nephropathy remained permanently on long-term hemodialysis, and their median survival was markedly shorter than that of patients with preserved renal function [6,7]. The introduction of bortezomib improved this outlook considerably, although the benefit was not uniform across patients.

Among the various renal lesions identifiable in myeloma, light-chain cast nephropathy predominates. Renal biopsy series have reported cast nephropathy in approximately 40–60% of specimens from patients with myeloma and kidney dysfunction; among dialysis-dependent patients, the proportion exceeds 70% in some cohorts [8,9,10]. Unlike AL amyloidosis or monoclonal immunoglobulin deposition disease, where renal recovery is generally infrequent and limited, cast nephropathy can be reversed if the nephrotoxic serum free-light-chain (sFLC) burden is reduced rapidly enough to prevent irreversible tubulointerstitial injury [6,11]. The therapeutic window is narrow, because experimental and clinical observations indicate that the longer cast-associated tubular injury persists, the lower the probability of functional recovery, as tubulointerstitial fibrosis and tubular atrophy progressively develop with sustained light-chain exposure [11,12,13]. In biopsy cohorts, approximately one-third of myeloma patients exhibit lesions other than cast nephropathy, which is why histology retains diagnostic value when the clinical presentation is ambiguous [14].

The kidney is the principal site of sFLC catabolism. Kappa monomers (22.5 kDa) and lambda dimers (45 kDa) are freely filtered through the glomerulus and degraded in the proximal tubule via megalin–cubilin-mediated endocytosis [15]. Once GFR declines, the circulating half-life of these molecules lengthens from 3–6 h to as much as 30 h in patients with advanced chronic kidney disease, creating a vicious cycle of escalating tubular exposure and accelerated cast formation [16,17]. While systemic chemotherapy remains the cornerstone of treatment by suppressing clonal sFLC production at its source, extracorporeal removal of circulating light chains has long been proposed as a synergistic adjunctive strategy to mitigate injury [18]. However, the effect of this approach on hard renal outcomes, and the dialysis membrane platform best suited to achieve it, remain surprisingly unclear. Previous reviews have typically focused on a single modality or on high-cutoff hemodialysis, whereas the present work starts from the therapeutic context in which any extracorporeal strategy is now applied. Since bortezomib-based induction and increasingly, anti-CD38-containing regimens lower sFLC within days, the question motivating this review is whether extracorporeal removal can still add incremental renal benefit when effective antimyeloma therapy is started without delay. The available membrane platforms are appraised in that light, and randomized and observational evidence is integrated with the pharmacokinetic and practical constraints that bear on dialysis modality selection in myeloma-associated AKI.

### Search Strategy and Appraisal of the Evidence

This article is a narrative review. PubMed/MEDLINE, Embase, and the Cochrane Central Register of Controlled Trials were searched without a lower date limit up to May 2026, combining terms for the condition (multiple myeloma, cast nephropathy, myeloma kidney, light chain, acute kidney injury, renal failure) with terms for the intervention (hemodialysis, high-cutoff, medium-cutoff, polymethylmethacrylate, hemodiafiltration, endogenous reinfusion, plasma exchange, plasmapheresis, extracorporeal); the reference lists of the retrieved articles, the International Myeloma Working Group (IMWG) recommendations, and ClinicalTrials.gov were screened in addition. English-language randomized trials, meta-analyses, cohort studies, case series, pharmacokinetic and membrane-characterization studies, and consensus documents on extracorporeal free-light-chain removal or dialysis modality selection in adults with myeloma-associated AKI were eligible when they reported free-light-chain kinetics, renal outcome, or safety; conference abstracts without a full publication and studies confined to monoclonal gammopathy-related lesions other than cast nephropathy were excluded. Given the heterogeneity of designs and response definitions, no quantitative synthesis was attempted.

## 2. Pathogenesis of Cast Nephropathy

From a mechanistic perspective, monoclonal light-chain-induced kidney injury occurs via two complementary processes: proximal tubular toxicity and distal cast-mediated obstruction. Under physiological conditions, filtered sFLCs undergo reabsorption by proximal tubular epithelial cells. However, when the filtered load exceeds the capacity of the megalin–cubilin endocytic system, excess intracellular light chains activate nuclear factor-κB (NF-κB) dependent inflammatory signaling through a Src-dependent pathway. This activates tubular apoptosis and induces the release of monocyte chemoattractant protein-1 (MCP-1), interleukin-6 (IL-6), and profibrotic cytokines, notably transforming growth factor-beta (TGF-β) [19,20,21]. Ying et al. demonstrated that monoclonal light chains can drive this proinflammatory and profibrotic injury even in the absence of overt cast formation, underscoring the direct nephrotoxicity of these proteins on the proximal tubule [21].

Downstream in the distal nephron, cast formation represents the primary driver of severe AKI. In a microperfusion study, Bence–Jones proteins coprecipitated with Tamm–Horsfall glycoprotein (uromodulin) in the lumen of the distal tubule, and light-chain casts are correspondingly identified at or beyond the thick ascending limb of the loop of Henle [22]. This interaction is largely determined by specific sequences within the complementarity-determining region 3 (CDR3) of the light-chain variable domain, which mediate binding to a discrete site on the uromodulin molecule identified by Huang and Sanders [23,24]. Because the propensity to bind uromodulin varies markedly among individual light chain clones, two patients with comparable sFLC concentrations may exhibit vastly different renal manifestations. The physicochemical character of the specific monoclonal light chain, rather than its circulating level alone, governs its nephrotoxic potential.

This intrinsic precipitation propensity can be significantly amplified by luminal factors. Volume depletion, hypercalcemia, non-steroidal anti-inflammatory drug (NSAID) use, loop diuretics, and contrast media can markedly reduce effective tubular flow and increase intratubular light chain concentration, thereby accelerating cast formation [22,25]. In the microperfusion model, furosemide increased cast-mediated tubular obstruction in a concentration-dependent manner, providing a rationale for the cautious use of loop diuretics in patients with suspected cast nephropathy [22].

While the clinical threshold above which cast nephropathy occurs is not absolute, observational series suggest that an sFLC concentration typically exceeding 500 mg/L, accompanied by proteinuria composed predominantly of light chains with a low albumin fraction, is strongly indicative of its presence [26,27]. Leung and colleagues showed that urinary albumin typically constitutes less than 10% of total proteinuria in cast nephropathy, versus more than 40% in AL amyloidosis or light-chain deposition disease [26]. The IMWG recommendations incorporate this diagnostic distinction; a renal biopsy should be considered in the presence of non-selective proteinuria with prominent albuminuria, or when involved sFLC concentrations are relatively low (e.g., <500 mg/L). These atypical patterns suggest alternative monoclonal gammopathy-related renal lesions or non-myeloma pathologies, requiring histological confirmation to guide appropriate management [5].

## 3. Bortezomib-Based Chemotherapy and Renal Recovery

### 3.1. The Pre-Proteasome Inhibitor Era

The introduction of proteasome inhibitors improved the chances of reversal of dialysis-dependent AKI in myeloma. Conventional regimens based on melphalan plus prednisone or vincristine–adriamycin–dexamethasone (VAD) induced hematologic responses too slowly to protect the kidneys from irreversible tubulointerstitial scarring, leaving renal recovery rates at approximately 25% [6,28].

### 3.2. The Bortezomib Era

The availability of bortezomib improved these prospects, and its pharmacology is well suited to the dialysis setting. Because bortezomib undergoes extensive hepatic oxidative metabolism, it does not require initial dose adjustment in patients with kidney impairment, including those receiving dialysis; in practice, it is generally administered after the dialysis session, when treatment and dialysis coincide. Hematologic responses are typically achieved within the first two cycles. Beyond rapid clonal suppression, bortezomib-mediated NF-κB inhibition may directly damp the downstream tubular inflammatory cascade triggered by filtered free light chains, a mechanism demonstrated in preclinical models but not established as a clinical renal benefit independent of clonal suppression [29,30,31].

Dimopoulos and colleagues analyzed outcomes in 83 newly diagnosed patients with severe renal impairment (eGFR < 30 mL/min/1.73 m^2^) treated with bortezomib-based regimens, of whom 31 (37%) required dialysis at baseline. According to those results, 15 of the 31 dialysis-requiring patients (48%) achieved dialysis independence at a median of 217 days, and bortezomib-based triple drug therapies produced a higher rate of dialysis discontinuation than bortezomib plus dexamethasone alone (57% vs. 35%), and the rate of renal response was higher (72% vs. 50%). The results also showed that rapid hematologic response within the first month predicted subsequent dialysis independence [32]. The role of novel agents in reversing renal impairment is further documented in a subsequent study by the same group, showing that patients treated with bortezomib-containing regimens had significantly higher rates of renal recovery than those treated with conventional chemotherapy, after adjustment for confounders [33]. Subgroup analyses from the VISTA trial showed that bortezomib plus melphalan and prednisone produced higher response rates and better survival than melphalan and prednisone across the renal function strata studied [34]. Because patients with serum creatinine above 2 mg/dL were excluded, these findings cannot be extrapolated to dialysis-dependent cast nephropathy.

To optimize bortezomib-based backbones, the MYRE study group randomized 184 patients with cast nephropathy not requiring immediate dialysis. Patients were assigned to receive either bortezomib–dexamethasone (Vd) or bortezomib–cyclophosphamide–dexamethasone (VCd). The study found no significant differences at 3 months in either renal response (44.6% vs. 51.1%; *p* = 0.46) or hematologic response (78.3% vs. 77.2%), with very good partial response or better in 39.1% and 51.1% (*p* = 0.14) and an involved sFLC of 500 mg/L or less after one cycle in 75.0% and 72.8% (*p* = 0.87) [35]. The trial used a protocol-mandated rescue after three cycles in patients with less than 50% free-light-chain reduction (cyclophosphamide added in the doublet arm, thalidomide added in the triplet arm). These findings indicate that, in non-dialysis-dependent cast nephropathy, adding cyclophosphamide upfront to a bortezomib–dexamethasone backbone did not improve either the speed of light-chain suppression or the depth of hematologic response at three months. More broadly, they are consistent with cohort observations associating the velocity of light-chain reduction in the first weeks with renal recovery, an association that describes the hematologic response to chemotherapy and carries no information, by itself, on the value of mechanical removal. In this acute phase, high-dose dexamethasone (commonly 40 mg daily for four days at initiation, reduced to 20 mg in patients over 75 yr) acts synergistically with the proteasome inhibitor. Dose attenuation during the initial emergency phase should be individualized, because excessive early reduction could theoretically slow clonal control, whereas full-dose dexamethasone may be poorly tolerated in frail or infection-prone patients [5,36].

### 3.3. The Daratumumab Era and Future Horizons

Recent updates from the IMWG highlight the emerging role of anti-CD38 monoclonal antibody-containing regimens in this setting. The DARE study, a prospective phase 2 trial of daratumumab plus dexamethasone, enrolled 38 heavily pretreated patients with relapsed or refractory myeloma (RRMM) and severe renal impairment (eGFR < 30 mL/min/1.73 m^2^). In that study, 17 of the 38 patients (44.7%) were receiving dialysis at enrolment, and the median baseline eGFR was 13.0 mL/min/1.73 m^2^. The hematologic overall response rate was 47.4% and the renal response rate 18.4% [37]. Similarly, the phase 2 GMMG-DANTE trial evaluated a different regimen, daratumumab plus bortezomib plus dexamethasone, in relapsed/refractory disease with severe renal impairment, including patients receiving hemodialysis [38]. The trial closed prematurely after enrolling 22 of the planned 36 patients but achieved its primary endpoint, with an overall response rate of 67% (14 of 21 evaluable patients), a renal response rate of 67%, and a median progression-free survival of 10.4 months [38]. Renal-response definitions differed across these anti-CD38 studies and are not directly comparable with the dialysis-independence endpoints of the MYRE and EuLITE trials.

Data from the frontline setting are more limited. A recent single-center retrospective cohort described 20 newly diagnosed patients treated with daratumumab-based therapy (daratumumab–VCd in 55% and daratumumab–Vd in 45%), with a median peak serum creatinine of 6.5 mg/dL and median peak involved sFLC levels of 6603 mg/L at diagnosis [39]. All 20 patients achieved a reduction in sFLCs of at least 50% within the first cycle, with a median time to this threshold of three days; 15 of 17 assessable patients (88%) achieved sFLC levels of 500 mg/L or less at a median of 14.5 days. The overall renal response at three months was 85%, comprising complete responses in 50%, partial responses in 10%, and minor responses in 25%. Nine patients required dialysis during their hospitalization. Two frail elderly patients aged 86 and 87 yr died within three months (from aspiration pneumonia and disease progression, respectively), leaving seven evaluable at three months; four of these seven (57%) were dialysis-independent at three months and six of seven (86%) at twelve months, with one patient remaining on dialysis at 12 months. These outcomes were achieved without plasmapheresis [39].

While these real-world outcomes are highly encouraging, important methodological caveats remain. These findings derive from a small, non-randomized, single-center retrospective cohort, rendering them inherently susceptible to selection bias and institutional expertise effects. The cohort also lacked biopsy confirmation of cast nephropathy, a critical distinction from the histologically verified populations of the landmark MYRE and EuLITE trials. Replication of such ultra-rapid sFLC kinetics across broader, unselected community settings requires prospective, randomized validation. Current evidence should not prematurely alter standard clinical practice.

A frontline signal comes from Japan, where Horigome and colleagues treated ten transplant-ineligible patients with newly diagnosed myeloma and severe renal impairment (eGFR ≤ 30 mL/min/1.73 m^2^, all stage III) with daratumumab, lenalidomide, and dexamethasone [40]. The hematologic response was 80%, with very good partial response or better in 50%; complete renal response occurred in 40%. The renal responders were also the deeper hematologic responders, and their progression-free survival was longer [40]. Study entry required only an eGFR ≤ 30 mL/min/1.73 m^2^, meaning these ten patients were not required to have biopsy-confirmed cast nephropathy or dialysis-dependent injury, rendering the relevance of these findings to dialysis-requiring patients purely indirect.

## 4. Plasma Exchange

The biological rationale for plasma exchange (PE) in myeloma-associated kidney injury rests on the premise that immunoglobulin light chains circulating as free monomers or dimers are accessible to mechanical removal by apheresis. This rationale is only partially supported by free-light-chain distribution. Free light chains are present at similar concentrations in serum, in the extravascular compartment, and in tissue edema fluid, so the intravascular compartment may hold only 15 to 20% of the total body burden, and reduction rates slow as extravascular stores re-equilibrate [41]. A two-compartment model of free-light-chain production, distribution, and metabolism predicted that plasma exchange would remove only 25% of the total amount over a three-week period, against 90% for extended high-cutoff hemodialysis [41]. Over the past four decades, three randomized controlled trials have evaluated this approach with conflicting results.

Zucchelli and colleagues randomized 29 patients with AKI due to MM and Bence–Jones proteinuria > 1 g/day to plasma exchange plus corticosteroids and a cytotoxic drug (with hemodialysis as needed, n = 15) or to peritoneal dialysis plus the same chemotherapy backbone (n = 14). In that study, 13 of 15 patients in the plasma exchange arm achieved renal recovery (serum creatinine typically <2.5 mg/dL), compared with 2 of 14 in the peritoneal dialysis arm, and one-year survival was 66% versus 28% (*p* < 0.01) [42]. The design varied two things at once; the experimental arm received plasma exchange with hemodialysis as needed, the control arm peritoneal dialysis. The survival gap is therefore as compatible with the difference in dialysis modality as with the apheresis itself.

Subsequently, Johnson et al. randomized 21 patients with active myeloma and progressive renal failure to forced diuresis and chemotherapy with or without plasmapheresis [43]. Plasmapheresis accelerated the decline in serum myeloma protein. Among the five patients who were oliguric and dialysis-dependent at presentation, only three recovered renal function, all of whom were in the plasmapheresis arm; however, the small sample size precluded robust statistical conclusions. Both trials were conducted before sFLC assays were introduced into clinical practice and before contemporary high-flux hemodialysis became standard. Biopsy confirmation of cast nephropathy was also not systematically obtained. These limitations constrain the applicability of the findings to current management.

The largest randomized trial was conducted by Clark and the Canadian Apheresis Group, who randomized 104 patients with AKI at myeloma presentation to 5–7 plasma exchanges of 50 mL/kg body weight using 5% human serum albumin as replacement fluid versus conventional therapy alone [44]. At six months, among the 97 of 104 patients who completed follow-up, there was no difference in the composite outcome of death, dialysis dependence, or a glomerular filtration rate below 30 mL/min/1.73 m^2^. Although this study is often cited as having settled the question, several limitations merit consideration. Biopsy confirmation of cast nephropathy was not required, heterogeneous chemotherapy regimens were used across the participating centers, and sFLC assays, now considered central to monitoring response, were not available at the time the trial was conducted.

A retrospective Mayo Clinic series subsequently suggested that plasma exchange might benefit the subset of patients with biopsy-proven cast nephropathy who achieved a greater than 50% reduction in sFLC, particularly when combined with bortezomib [45]. Burnette, Leung, and Rajkumar reported on 14 patients treated with bortezomib plus plasma exchange: 12 of 14 achieved complete or partial renal response by six months, but the conclusions were limited by the lack of a control group [46].

Contemporary real-world data support this position. In a ten-year Turkish retrospective cohort of 71 patients with myeloma-related cast nephropathy treated between 2013 and 2023, renal response rates were similar between the 30 patients who received plasma exchange in addition to antimyeloma therapy and the 41 who received antimyeloma therapy alone (40% versus 36.6% at the end of the first cycle, and 60.0% versus 48.7% at the end of cycles four to six), and plasma exchange was not an independent predictor of renal improvement on multivariable analysis [47]. A potential pharmacokinetic interaction is a further consideration now that anti-CD38 therapy is standard, because daratumumab is an IgG monoclonal antibody; plasma exchange performed close to drug administration may theoretically remove part of the circulating drug, although the magnitude and clinical relevance of this interaction in myeloma cast nephropathy have not been established [39]. That argument does not extend to high-cutoff membranes, whose pores exclude a molecule of that size.

The IMWG recommendations do not endorse plasma exchange as standard care and explicitly state that mechanical approaches do not increase overall survival [5]. In current clinical practice, therapeutic plasma exchange is not supported for routine use. Exceptional use remains center- and case-dependent, but no clinical or biochemical criterion has been validated to identify a subgroup with improved renal outcomes [5,27].

## 5. High-Cutoff Hemodialysis: Rationale, Evidence, and Limitations

### 5.1. Pharmacokinetic Basis

Conventional dialyzers carry a nominal cutoff of approximately 20 kDa, which limits clearance of kappa monomers (≈22.5 kDa) and lambda dimers (≈45 kDa) [41]. Hutchison and colleagues screened seven dialyzers and identified the Gambro HCO 1100, with a reported cutoff of 45 kDa, as the most efficient; among dialyzers with a cutoff up to 20 kDa, binding to the membrane rather than permeation was the principal clearance mechanism [41]. In vivo, sFLC clearance rates of 10 to 40 mL/min were achieved, clearance correlating with dialysate flow rate as 10.8 mL/min for a dialysate flow of 300 mL/min and 19.3 mL/min at 500 mL/min [41]. Albumin leakage is inherent to a membrane whose cutoff approaches the molecular weight of albumin (65 kDa); replacement of 20 to 40 g per session was required, and serum albumin fell by a mean of 3.9 g/L across sessions [41]. In a subsequent optimization study in 14 patients, two dialyzers in series raised clearance from 19 to 47 mL/min for kappa and from 15.3 to 35.5 mL/min for lambda, kappa clearance exceeding lambda throughout, while albumin replacement requirements over 8 h of dialysis rose from 12 g with a single dialyzer to 45 g with two in series [48]. Clearance declined within each session, in a pattern attributable to the plasma protein layer that adsorbs onto the membrane on contact with blood and reduces its effective permeability [49]. Ultrafiltration during hemodialysis increased kappa clearance but not lambda, whereas hemodiafiltration raised the clearance of both isotypes, from 19 to 32 mL/min for kappa and from 15 to 20 mL/min for lambda [48]. The convective mode also amplifies albumin loss; with a 2.1-m^2^ high-cutoff dialyzer, median loss rose from 21 g per session in hemodialysis to 63 g per session in hemodiafiltration [50]. The first clinical cohort to apply this strategy with chemotherapy was reported by Hutchison and colleagues. In 19 patients with biopsy-proven cast nephropathy and dialysis-dependent AKI, 14 (74%) became dialysis independent. Sustained early reductions in sFLC were observed in 13 patients, all of whom recovered renal function at a median of 27 days, with the remaining responder regaining renal function only at 105 days; of the six patients in whom chemotherapy was interrupted because of early infections, only one regained renal function [51]. A parallel German cohort by Heyne and colleagues, also including 19 dialysis-dependent patients (10 newly diagnosed and 9 relapsed/refractory), reported sustained renal recovery in 14 patients with a median time of 15 days to dialysis independence; on multivariate analysis, the duration of AKI before initiation of therapy was the only independent predictor of recovery [52].

A separate pharmacokinetic concern involves the potential clearance of antimyeloma drugs during high-cutoff sessions. An in vitro study by Krieter et al. demonstrated measurable clearance of thalidomide, bortezomib, and dexamethasone across HCO membranes, with thalidomide showing the greatest removal [53]. However, the clinical relevance of these findings is debated. Bortezomib has high protein binding (approximately 83%), which would be expected to limit its transmembrane passage in vivo. In vitro studies using spiked solutions may overestimate actual clearance during clinical dialysis. Formal pharmacokinetic studies during HCO hemodialysis in patients receiving bortezomib are lacking. In principle, drug dosing may be timed after dialysis when feasible to minimize any potential therapeutic losses, though this recommendation rests on theoretical rather than clinical evidence.

### 5.2. The MYRE Trial

One of the cardinal studies for understanding the value of high-cutoff (HCO) hemodialysis in MM is the MYRE trial. MYRE enrolled 98 patients with biopsy-proven cast nephropathy and dialysis-dependent AKI, at 48 French centers, randomizing the patients between intensive high-cutoff and conventional high-flux dialysis on a shared bortezomib–dexamethasone backbone [54]. A 4- to 15-day screening phase preceded randomization; dehydration and other precipitating factors were corrected, hypercalcemia was treated, and all patients received 4 days of high-dose dexamethasone or intravenous methylprednisolone. Only those still requiring dialysis after this run-in, with biopsy confirmation and secreting myeloma, entered the trial proper. Both arms received eight 5-h sessions over the first 10 days and then thrice-weekly treatment until three chemotherapy cycles were complete, with the high-cutoff arm using the 2.1-m^2^ Theralite dialyzer (Gambro). Per-session free-light-chain removal was markedly greater on high-cutoff; median κ reduction after the first dialysis was 77% (IQR 67–86) versus 35% (IQR 19–48), while λ reduction was 63% (IQR 52–72) versus 18% (IQR 6–56), both *p* < 0.001. This biochemical advantage did not carry over to the primary endpoint. In the modified intention-to-treat population (n = 94), hemodialysis independence at three months stood at 41.3% versus 33.3%, a between-group difference of 8.0% (95% CI −12.0 to 27.9; *p* = 0.42), and the trial missed its prespecified target. A between-group difference did emerge at later time points; dialysis independence reached 56.5% versus 35.4% at six months (difference 21.1%, 95% CI 0.9 to 41.3; OR 2.37, 95% CI 1.03–5.44; *p* = 0.04), and 60.9% versus 37.5% at twelve months (difference 23.4%, 95% CI 3.2 to 43.5; OR 2.59, 95% CI 1.13–5.96; *p* = 0.02), although the investigators framed these study results as exploratory in light of the multiple-comparison problem. Renal recovery is biologically delayed relative to free-light-chain reduction, since tubular repair and the resolution of established casts proceed over weeks to months after the circulating burden has fallen, so an endpoint set at three months may register the biochemical intervention before its renal consequence is complete. This offers a rationale for the later separation, though it cannot distinguish a genuine delayed benefit from a chance finding among multiple comparisons. On multivariable analysis in the MYRE report, the predictors of dialysis independence within 12 months, after adjustment for myeloma subtype and post-treatment free-light-chain concentration, were whole-immunoglobulin (versus light-chain-only) disease (OR 2.75, 95% CI 1.11–6.80; *p* = 0.03), an involved sFLC ≤ 500 mg/L after one cycle (OR 2.51, 95% CI 1.00–6.33; *p* = 0.049), and randomization to the high-cutoff arm itself (OR 2.78, 95% CI 1.13–6.80; *p* = 0.03). Overall survival was indistinguishable (HR 0.76, 95% CI 0.36–1.58; log-rank *p* = 0.46). The hematologic response at three months, by contrast, diverged sharply: 89.1% versus 62.5% (difference 26.6%, 95% CI 9.8 to 43.4; OR 4.9, 95% CI 1.6–14.7; *p* = 0.003). Since the antimyeloma regimen was shared, the investigators themselves attributed the gap to more efficient free-light-chain removal on dialysis. This interpretation is problematic because hematologic response in light-chain-only disease is defined on sFLC concentrations, the very quantity high-cutoff dialysis removes, so mechanical lowering of sFLC may have inflated the apparent hematologic response rate, particularly in light-chain-only disease, although the magnitude of this effect cannot be determined. On balance, tolerance was acceptable, although post-dialysis albumin infusion for predialysis albumin < 25 g/L was needed in 41% of high-cutoff sessions against 4% of conventional ones [54].

### 5.3. The EuLITE Trial

Another landmark trial is EuLITE, in which 90 patients with newly diagnosed myeloma, biopsy-confirmed cast nephropathy, and dialysis-dependent AKI were enrolled across 16 centers in the United Kingdom and Germany [55]. Randomization was 1:1 (43 to high-cutoff, 47 to high-flux) without a run-in phase. The high-cutoff regimen used two 1.1-m^2^ HCO-1100 dialyzers in series (combined surface area 2.2 m^2^): a 6 h first session followed by seven 8 h sessions during the first 10 days, with treatment continued for up to 3 months. MYRE, by contrast, used 5 h sessions on a single 2.1 m^2^ Theralite dialyzer. The authors calculated that EuLITE delivered roughly 40% more total high-cutoff dialysis time in the first 10 days, and persistently more thereafter. Chemotherapy regimens also differed. EuLITE delivered a triplet of reduced-dose bortezomib (1 mg/m^2^ rather than the standard 1.3 mg/m^2^), doxorubicin, and dexamethasone; MYRE used a full-dose bortezomib–dexamethasone doublet (1.3 mg/m^2^), with cyclophosphamide added only if needed. The bortezomib dose was lower in EuLITE, but doxorubicin may have amplified myelosuppression and infection risk. Dialysis independence at 90 days was equivalent, 24/43 (56%) versus 24/47 (51%), RR 1.09 (95% CI 0.74–1.61; *p* = 0.81), and the hazard ratio for time to dialysis independence stood at 0.91 (95% CI 0.54–1.54; *p* = 0.72), unchanged after age adjustment [55]. No delayed benefit in dialysis independence emerged at any later assessed time point. Over 24 months, 25/43 (58%) in the high-cutoff arm and 31/47 (66%) in the high-flux arm reached dialysis independence; at 12 months, myeloma response was lower under high cutoff (42% vs. 68%; RR 0.62, 95% CI 0.41–0.92; *p* = 0.022). Moreover, the high-cutoff regimen was associated with a less favorable safety profile, with a higher burden of serious adverse events, particularly infections. Lung infections within the first 90 days were concentrated in the high-cutoff arm: 14 episodes across 13/43 patients (30%) against 3 episodes in 3/46 controls (7%). In a non-prespecified post hoc analysis, the relative risk of at least one lung infection was 4.64 (95% CI 1.42–15.20; *p* = 0.008). Reportedly, 5 of these 13 high-cutoff recipients later died (3 from myeloma progression, 2 from infection), against none of the three controls with an early lung infection. Treatment tolerability followed the same direction: 9/43 (21%) discontinued high-cutoff treatment versus 2/46 (4%) on high flux. By two years, 16/43 (37%) in the high-cutoff arm had died, against 9/47 (19%) on high flux; the prespecified unadjusted analysis gave a survival hazard ratio of 2.17 (95% CI 0.96–4.91; log-rank *p* = 0.058), and a pre-planned sensitivity analysis adjusting for age crossed significance (HR 2.63, 95% CI 1.13–6.15; *p* = 0.026, reported as *p* = 0.03. Causes of death distributed similarly across arms (advanced myeloma 7/16 vs. 4/9; infection 5/16 vs. 3/9), prompting the authors to observe that no single dominant mortality driver could anchor a treatment-related effect, given the small numbers. In accompanying commentaries, Sallée and Burtey called these results the ‘dusk’ of high-cutoff hemodialysis for this indication; Bridoux, Chevret, and Fermand argued that protocol and selection differences might still justify further investigation in selected settings [56,57].

### 5.4. Reconciling the Two Trials: A Critical Appraisal

The discordance between MYRE and EuLITE has several potential explanations, but before examining them, it is worth emphasizing what both trials actually demonstrated. Neither trial met its primary endpoint. Both were relatively small (MYRE enrolled 98 patients, EuLITE 90) and in both, wide confidence intervals were reported around their primary estimates, meaning that clinically relevant treatment effects in either direction could not be excluded. The MYRE investigators themselves acknowledged underpowering and framed their positive secondary analyses at 6 and 12 months as exploratory because of the potential for type I error from multiple comparisons [54]. The EuLITE investigators likewise observed that the trial was powered on the basis of a large treatment effect (an improvement in renal recovery from 25% to 55%) and had low power to detect a more modest effect [55].

Several concrete design differences between the two trials may have contributed to the divergent findings, and many of these were noted by the investigators in each trial. MYRE incorporated a 4-to-15-day screening phase during which dehydration was corrected, hypercalcemia treated, and high-dose steroids administered before randomization; only patients who still required hemodialysis after this period were enrolled. EuLITE did not include such a screening phase. This is likely to have populated MYRE with patients with established, non-reversible cast-nephropathy-driven kidney injury and may explain the delayed signal of benefit observed in MYRE at 6 and 12 months. As the investigators themselves proposed, supportive measures and steroids alone could lead to early recovery in a subset of patients who would otherwise have been randomized. The chemotherapy regimen also differed substantially. MYRE used a bortezomib–dexamethasone doublet, with cyclophosphamide reinforcement after three cycles only in patients with insufficient hematologic response. EuLITE used a bortezomib–doxorubicin–dexamethasone triplet with a reduced bortezomib dose of 1 mg/m^2^, a combination chosen for its expected efficacy but plausibly also contributing to greater myelosuppression and infectious risk. The reduced bortezomib dose warrants emphasis in its own right. Because the speed and depth of the hematologic response drive renal recovery, a regimen delivering less proteasome inhibition than the standard 1.3 mg/m^2^ may have constrained renal recovery in both arms, and with it, the margin within which enhanced light-chain removal could have declared itself. The trial therefore tested high-cutoff dialysis against a chemotherapy that was not the contemporary standard. The dialysis prescription differed as well. EuLITE delivered 6 h first sessions and 8 h subsequent sessions with two 1.1-m^2^ HCO-1100 dialyzers in series (combined surface area 2.2 m^2^), whereas MYRE delivered 5 h sessions with a single 2.1-m^2^ Theralite dialyzer. The EuLITE investigators calculated that their trial delivered approximately 40% more high-cutoff dialysis exposure in the first 10 days and considerably more thereafter. These design differences cumulatively produced a more aggressive intervention in EuLITE, which may have contributed to greater albumin and immunoglobulin depletion, higher infection risk, and the significantly worse 12-month myeloma response and age-adjusted overall survival observed in the high-cutoff arm, although this sequence remains a plausible rather than a proven explanation. The 21% treatment discontinuation rate in that arm, compared with 4% in controls, adds procedural tolerability to the same picture [54,55].

A related point concerns what MYRE actually proved. With high-cutoff dialysis, per-session κ clearance more than doubled, and hematologic response at three months reached 89.1% compared with 62.5% with conventional dialysis, confirming the biochemical effect high-cutoff dialysis was designed to produce [54]. However, this biochemical and hematologic advantage was not accompanied by a superior primary renal endpoint. Moreover, with effective chemotherapy administered concurrently, the rate-limiting step for renal recovery may primarily reflect the extent of tubulointerstitial injury already established at randomization rather than the speed of circulating light chain removal with high cutoff dialysis, although neither trial was powered to test this directly. Once cast-mediated damage has progressed beyond the point of reversibility, faster dialytic clearance is unlikely to restore renal function. Conversely, when damage remains reversible, antimyeloma therapy alone may suffice to promote renal recovery.

Neither trial was powered for subgroup analysis. The existence of specific populations deriving a net benefit from high-cutoff dialysis remains untested in any rigorous fashion. These include anuric patients at presentation, those with particularly high circulating free-light-chain levels, those whose light chains fail to fall rapidly with chemotherapy alone, and patients with lambda-restricted disease whose dimeric light chains are less readily cleared [56,57]. Each of these candidate subgroups should accordingly be read as a hypothesis for a future trial, and no threshold of free light chain concentration, kinetics or urine output has been validated to define any of them.

A meta-analysis by Tarragón and colleagues pooled two randomized trials and three observational studies (276 patients) and found no survival benefit for high-cutoff hemodialysis at 12 months (RR 1.02, 95% CI 0.76–1.35) or at end of follow-up (RR 1.32, 95% CI 0.71–2.45) [58]. Within the randomized subset (188 patients), dialysis independence was no different at 6 months (RR 1.19, 95% CI 0.68–2.06) or 12 months (RR 1.14, 95% CI 0.58–2.26), although heterogeneity behind both estimates was substantial (I^2^ 71.8% and 81.3%), which is unsurprising given the discordance of the two trials being pooled [58]. The pooled randomized evidence establishes neither a survival nor a renal benefit, though the point estimates for dialysis independence lie above unity throughout and the authors read them as a non-significant trend favoring high-cutoff dialysis [58]. A registered randomized trial directly comparing high-cutoff, medium-cutoff, and conventional high-flux membranes beyond the completed MYRE and EuLITE studies had not appeared on ClinicalTrials.gov as of May 2026.

Two observational studies included in the Tarragón analysis warrant explicit consideration. Gerth et al., in a retrospective case-control study of 59 dialysis-dependent patients, reported renal recovery in 64.3% of the 42 patients treated with high-cutoff hemodialysis versus 29.4% of the 17 treated with conventional hemodialysis (*p* = 0.014), with high-cutoff dialysis remaining an independent predictor on multivariable analysis (adjusted OR 6.1; 95% CI 1.5–24.5) [59]. A contemporaneous Swiss single-center series by Curti et al. reported a non-significant trend toward faster renal recovery with high-cutoff dialysis [60]. Neither study matched MYRE or EuLITE in methodological rigor, and both are subject to the selection biases inherent to non-randomized comparisons in a rapidly evolving therapeutic landscape. The randomized trials of extracorporeal free-light-chain removal are summarized in Table 1.

## 6. Alternative Extracorporeal Strategies: A Comparative Appraisal of Membrane Platforms

Beyond high-cutoff hemodialysis, several alternative extracorporeal platforms have been investigated for light-chain removal, each characterized by differing trade-offs between clearance efficiency, albumin preservation, and logistical complexity. A recent comparative review by Heybeli and colleagues synthesized the available data across medium-cutoff membranes, polymethylmethacrylate adsorptive dialyzers, hemodiafiltration with endogenous reinfusion, and membrane-based adsorption or apheresis techniques, highlighting that none of these approaches has yet been tested against conventional hemodialysis in adequately powered randomized trials. As a result, their comparative positioning currently rests largely on clearance data, mechanistic considerations, and small observational series [61]. The level of evidence therefore differs by an order of magnitude from that available for high-cutoff dialysis; for medium-cutoff membranes, it consists of crossover clearance studies in patients without myeloma, one case series of three patients, and two retrospective cohorts; for PMMA membranes, it includes one crossover clearance study and two retrospective cohorts; for HFR and HFR-Supra, it comprises one crossover clearance study and two observational series of 13 and 9 patients. None of these studies had a randomized comparator, most were single-center, biopsy confirmation was reported inconsistently, and the chemotherapy backbone varied within as well as between series. The reduction ratios reported below and in Table 2 accordingly document that each platform removes free light chains; they carry no information on renal outcome relative to conventional high-flux dialysis, and the sections that follow should be read as an account of what has been observed, with the comparative clinical positioning of these platforms regarded as unresolved.

### 6.1. Medium-Cutoff Membranes

Medium-cutoff (MCO) membranes were developed to deliver the expanded toxin removal capability of high-cutoff membranes while adequately retaining albumin, so that they remain suitable for conventional treatment schedules [62]. This class, introduced as medium-cutoff, is increasingly designated high-retention-onset, in recognition that the retention onset rather than the cutoff is its distinguishing parameter; the original term is retained here for consistency with the cited literature. Their characterization rests on two parameters rather than one: the molecular weight retention onset, at which the sieving coefficient reaches 0.9, and the molecular weight cutoff, at which it falls to 0.1 [62]. Both shift substantially once the membrane meets blood. Boschetti-de-Fierro and colleagues characterized four MCO prototype membranes by dextran sieving against a conventional high-flux membrane (Revaclear) and a high-cutoff membrane (Theralite), before and after 40 min of blood exposure [62]. After blood contact, retention onset ranged from 5.0 to 6.8 kDa and cutoff from 18.1 to 25 kDa across the MCO prototypes, against 4.4 and 14.2 kDa for the high-flux membrane and 8.1 and 40 kDa for Theralite, whose pristine cutoff of 300 kDa thus falls by close to an order of magnitude in operation [62]. Because the protein layer has already formed by 40 min of blood contact, the membrane doing the work through most of a session is a fouled one [49,62]; fouling continues to accrue thereafter, and clearance falls further as the session proceeds [48]. Cutoff figures quoted for these membranes are therefore not interchangeable, and their comparison with the molecular weight of free light chains is unsafe for a further reason; dextran sieves differently from globular proteins, the glomerular barrier being more permeable to dextrans than to ficolls and more permeable to ficolls than to globular proteins [62]. A comparison by hydrodynamic radius comes closer, with the caveat that the pore radii are themselves derived from dextran sieving and carry the same conformational bias; so, the exercise remains indicative. Boschetti-de-Fierro reported radii of 2.3 nm for monomeric free light chains, predominantly kappa, 2.8 nm for the dimeric form, predominantly lambda, and 3.51 nm for albumin, against effective pore radii after blood contact of 2.68 nm for the conventional high-flux membrane, 3.0 to 3.5 nm across the MCO prototypes, and 4.3 nm for Theralite [62]. Three tendencies follow, on the understanding that sieving decays continuously as the solute radius approaches the pore radius, and that a membrane offers a distribution of pore sizes rather than a single one, so that passage of the largest solutes is carried by the upper tail of that distribution. The working pore of a conventional high-flux membrane falls between kappa and lambda, which is consistent with the isotype asymmetry recurring across the series discussed below. The least permeable MCO membrane sits at 3.0 nm, above lambda and below albumin, a window the authors judged sufficient for removal of large uremic toxins up to lambda free light chains; that the observed lambda reduction ratios exceed what those dimensions would predict on a single-pore reading is itself an argument for the narrower pore distribution that defines the class. The high-cutoff membrane sits above albumin, resulting in loss of albumin [62]. These dimensions describe monomers and dimers. Free light chains also polymerize to a variable degree, which the original pharmacokinetic work identified as an unmeasured source of between-patient variation in clearance [41]; polymeric species sit outside the window of any of these membranes. In two randomized, open-label, crossover pilot studies enrolling 39 prevalent hemodialysis patients, Kirsch and colleagues compared MCO dialyzers, including the Theranova 400, against a high-flux dialyzer in hemodialysis and against high-volume hemodiafiltration, with lambda free-light-chain clearance as the primary outcome [63]. Per-session reduction ratios with MCO reached 66.3 to 70.4% for kappa and 42.5 to 51.5% for lambda, against 36.4 and 12.9% with high-flux hemodialysis (each *p* < 0.001) [63]. Against high-volume hemodiafiltration, MCO was superior for lambda (48.1 and 52.7% versus 37.9%; *p* < 0.001) and no different for kappa [63]. The studies characterized membrane clearance across a solute size range of 11.6 to 45 kDa [63]. Albumin removal was not negligible; median loss per treatment was 2.9 g with the Theranova 400 and rose to 4.8 and 7.3 g with the two more permeable prototypes, against 0.2 g with high-flux hemodialysis and 0.4 g with hemodiafiltration [63]. Participants were prevalent dialysis patients with a normal kappa-to-lambda ratio and no history of monoclonal gammopathy; these figures therefore describe polyclonal free light chains at concentrations far below those of cast nephropathy.

In the specific context of myeloma cast nephropathy, Cazorla López and colleagues described three patients with kappa light-chain cast nephropathy treated with 6 h MCO hemodialysis sessions (Theranova 500) combined with bortezomib–dexamethasone [64]. Per-session kappa free-light-chain reductions averaged 44.8 ± 19.5%, without clinically significant albumin loss. All three patients recovered renal function and became dialysis-independent. A larger retrospective cohort from Munich included 60 evaluable patients treated between 2015 and 2021, of whom 55 received MCO and 5 received HCO as a technical control. Over the whole dialysis treatment period, median reductions with MCO were 58% (IQR 30–80) for κ and 39% (IQR 22–72) for λ, against 84% (IQR 53–94) and 33% (IQR 24–60) with HCO. Per-session data were reported separately, for MCO only, in a subset of 10 patients: median κ reduction 70% (IQR 39–81) and λ reduction 37% (IQR 19–50). In that study, 34 of the 60 patients were off dialysis at one year, among 37 surviving to follow-up. The study was non-randomized and markedly unbalanced between arms, the chemotherapy backbone was adapted according to response, and the authors could not separate the effect of chemotherapy from that of extracorporeal removal, so causal inference is limited [65]. Both groups received a mean of eight 4 h sessions, substantially shorter than the 5–8 h high-cutoff protocols of MYRE and EuLITE, with implications for treatment burden.

A systematic review by Koniman and colleagues identified only case series data for medium-cutoff dialyzers in this setting and concluded that, while the available evidence is encouraging, large-scale randomized trials are required before formal recommendations can be made [66]. From a practical standpoint, MCO membranes are compatible with standard dialysis platforms and do not typically require albumin replacement. They may also facilitate ultrafiltration more readily than HCO membranes, where efforts to limit albumin loss can constrain fluid removal in practice [64,66]. These logistical advantages make MCO membranes operationally attractive and warrant prospective evaluation, but their clinical effectiveness in cast nephropathy remains unproven.

Skerget and colleagues recently reported a Slovenian cohort, treated between 2020 and 2023, in which the membrane was matched to the isotype, using MCO dialysis for kappa-restricted disease and HCO for lambda, starting both dialysis and antimyeloma therapy on the day of diagnosis [67]. On the MCO membrane, kappa clearance matched what HCO achieved for lambda. Free light chains fell from a median of 9630 mg/L to 2400 mg/L at day 7, 1083 mg/L at day 14, and 370 mg/L at day 30, after a median of four sessions. The hematologic response at three months reached 87%, with very good partial response or better in 35% and a renal response in 79%. Survival diverged sharply with early kinetics; patients whose sFLC fell by at least 70% within the first week had a median overall survival of 82.5 months, against 23.2 for the rest [67]. The series was retrospective and had no control arm, and, as in earlier cohorts, the biochemical response was more pronounced than the renal recovery. The practical implications are correspondingly limited; a short course of MCO dialysis is readily combined with current induction regimens, and the speed of the early biochemical response identified the patients likely to recover, whichever membrane had been used.

### 6.2. Polymethylmethacrylate Adsorptive Membranes

Polymethylmethacrylate (PMMA) membranes, notably the Toray Filtryzer BK-F (Toray Industries, Tokyo, Japan; surface area 2.1 m^2^), remove proteins by diffusion and convection and, in addition, by adsorption onto the hydrophobic membrane surface [68,69]. Published cutoff values for this dialyzer are discordant, reported as 20 kDa in one crossover study and 60 kDa in a myeloma cohort [69,70], which illustrates how unreliable the parameter is across this literature; for either figure, the adsorptive properties enable removal of molecules beyond the nominal permeation limit. This is particularly relevant for kappa free light chains (≈22.5 kDa), for which removal is facilitated by both permeation and adsorption, and for lambda chains, which may be removed in part through adsorption despite their larger dimeric structure (≈45 kDa).

In a crossover study comparing PMMA, hemodiafiltration with endogenous reinfusion (HFR), and conventional polysulfone membranes in 20 chronic hemodialysis patients, PMMA achieved per-session reduction ratios of 55.3 ± 3.4% for kappa and 37.8 ± 17.3% for lambda, compared with 25.7 ± 8.3% and 14.0 ± 3.9%, respectively, for conventional polysulfone membranes [69].

Sens and colleagues reported a single-center cohort of 17 patients with dialysis-dependent cast nephropathy treated with intensive hemodialysis using an adsorbent polymethylmethacrylate membrane, 88% of them on a bortezomib-based regimen [70]. Each session lasted 6 h and consumed two BK-F 2.1 dialyzers, the first replaced after 3 h to forestall membrane saturation, and sessions were given six times weekly until pre-dialysis free light chains fell below 200 mg/L or for a maximum of three weeks; that target was reached in only 41% of patients [70]. In that study, 12 of 17 patients (71%) achieved renal recovery, at a median of 23 days. Median free-light-chain reduction was 76% by day 12 and 84.5% by day 21, each associated with renal recovery (*p* = 0.022 and *p* = 0.003), and patients achieving a reduction exceeding 50% by day 21 had lower mortality (HR 0.10; 95% CI 0.02–0.63) [70]. No patient required albumin replacement over the treatment period, against albumin supplementation in more than a third of high-cutoff sessions in MYRE [70].

The dual-PMMA (DELETE) system, described by Santoro and colleagues, employs two BK-F dialyzers in series to enhance adsorptive capacity while maintaining adequate small-solute clearance [68]. This approach has primarily been applied in chronic dialysis patients with persistently elevated free light chains, and its role in acute cast nephropathy has not been formally evaluated.

A Spanish multicenter retrospective study of 25 patients with severe AKI secondary to myeloma compared intensive hemodialysis using PMMA or HFR (n = 17) with conventional hemodialysis (n = 8); renal recovery occurred in 14 of 25 patients overall (56%) and was associated with the use of an intensive dialysis strategy; the study did not compare PMMA against HFR, and the small sample size and non-randomized design limit interpretation [71]. All clinical evidence for PMMA membranes in cast nephropathy derives from observational cohorts, and no randomized comparison against conventional dialysis has been undertaken.

### 6.3. Hemodiafiltration with Endogenous Reinfusion (HFR and HFR-Supra)

The HFR family of systems (Bellco/Medtronic, Mirandola, Italy) employs a two-stage circuit in which an endogenous ultrafiltrate, generated by a high-permeability hemofilter, passes through a styrenic resin sorbent cartridge that adsorbs middle molecules including free light chains; the purified ultrafiltrate is then reinfused into the blood, which proceeds to a downstream low-flux polyphenylene filter (surface area 1.7 m^2^) for diffusive clearance and net ultrafiltration [69,72]. Two generations are described in the literature, although the terms “HFR” and “HFR-Supra” are at times used interchangeably. The original HFR couples a polyphenylene hemofilter (cutoff approximately 35 kDa, surface area 0.7 m^2^) and a styrenic resin cartridge with the downstream low-flux filter. The newer HFR-Supra replaces the first-stage filter with a super-high-flux Synclear 0.2 membrane, of 0.7 m^2^ surface area and a reported cutoff of 45 kDa, and pairs it with a higher-capacity sorbent cartridge, so that the ultrafiltrate carries higher concentrations of solutes overlapping in size with free light chain dimers [73,74]. The resin is a macroporous neutral styrenic material [69,73].

The principal advantage of this approach is that free-light-chain removal occurs predominantly in the ultrafiltrate phase, while albumin, which is only minimally filtered across the first membrane, is largely preserved. In a crossover study by Donati et al., conducted in chronic hemodialysis patients without myeloma, HFR achieved kappa reduction ratios of 44.1 ± 4.3% and lambda reduction ratios of 30.3 ± 2.9%, without significant changes in albumin or C-reactive protein (CRP) levels [69].

The HFR-Supra configuration was evaluated in a single-center Italian series of 13 patients with myeloma and stage III AKI (10 with biopsy-confirmed cast nephropathy), treated with daily HFR-Supra sessions (mean 10 per patient) alongside various chemotherapy regimens [75]. Median lambda free light chains at diagnosis were 8586 mg/L and median serum creatinine was 7.5 mg/dL. Cumulative reductions over the treatment cycle reached 85% for kappa and 40% for lambda, consistent with the molecular weight differential between kappa monomers and lambda dimers. Renal recovery was observed in 6 of 13 patients and was attributed by the authors to the combined effect of chemotherapy and extracorporeal removal. The absence of albumin replacement requirements and the ability to perform ultrafiltration during treatment were highlighted as practical advantages over high-cutoff hemodialysis [75].

Pendón-Ruiz de Mier et al., from Córdoba, in a separate observational series of nine patients (five with kappa- and four with lambda-restricted disease, according to the involved light chain), confirmed that hemodiafiltration with ultrafiltrate regeneration reduced free light chains without clinically relevant albumin loss, reporting kappa and lambda reduction ratios of 57.6 ± 10% and 33.5 ± 25%, respectively, with renal recovery in 33.3% of patients [72]. The theoretical profile of HFR/HFR-Supra (combined high-permeability filtration with adsorptive removal, endogenous reinfusion to limit albumin loss, and compatibility with net ultrafiltration) is attractive [74], but no controlled data exist for either configuration in cast nephropathy. A prospective interventional study of HFR-Supra in myeloma has been registered (ClinicalTrials.gov NCT05429515), although the registry record has not been updated since 2022 and no results are available; until such data emerge, any extrapolation from the limited observational experience should be made with caution.

Conventional online hemodiafiltration offers a third route that is widely available and spares albumin. In a single patient studied by Kamido and colleagues, per-treatment reduction ratios were 16% with high-flux hemodialysis and 20% to 31% with online hemodiafiltration, against 75% for plasma exchange [76]. Kidney function did not return despite hematologic remission; the biopsy showed extensive interstitial fibrosis with a heavy cast load, and treatment had begun late [76]. In this case, the efficiency of clearance was immaterial, because the interstitial damage was already established.

### 6.4. Polyester Polymer Alloy (PEPA) Membranes

Polyester polymer alloy (PEPA) membranes, marketed by Nikkiso as the FLX series, combine polyethersulfone and polyarylate to produce an adsorptive surface with middle-molecule removal characteristics that exceed those of standard polysulfone membranes [77]. Donati and colleagues noted that polysulfone membranes exhibit measurable intrinsic adsorptive properties, which are further enhanced in newer polyethersulfone and PEPA formulations [69]. Published clinical data on PEPA membranes in myeloma cast nephropathy are scarce and limited to anecdotal reports, with no dedicated studies to date. In the authors’ opinion, the membrane’s middle-molecule clearance profile and limited albumin loss might merit investigation in this setting, but its role in cast nephropathy is presently undefined and no recommendation can be made. For the same reason, PEPA membranes are discussed here but are not listed in the comparative table, which is restricted to platforms with reported free-light-chain removal data.

### 6.5. Conventional High-Flux Hemodialysis and Polysulfone Membranes

Conventional high-flux polysulfone membranes remove a modest fraction of circulating free light chains per session. In the control arm of MYRE, median reduction after the first dialysis was 35% (IQR 19–48) for kappa and 18% (IQR 6–56) for lambda [54]; these figures were closely matched in prevalent dialysis patients without myeloma, where a high-flux dialyzer achieved 36.4% for kappa and 12.9% for lambda [63]. A low-flux polysulfone membrane of 11.5 kDa cutoff performed less well for kappa and comparably for lambda, at 25.7 ± 8.3% and 14.0 ± 3.9% [69]. Widening the pore across the conventional range therefore recovers kappa while leaving lambda largely excluded on either side of that step. Convective transport can remove solutes that diffusion across the pore does not; hemodiafiltration through a high-flux membrane raised lambda clearance from 15 to 20 mL/min [48]. In both the MYRE and EuLITE trials, the conventional high-flux control arms nonetheless achieved dialysis independence rates of approximately 33–51% at three months, apparently reflecting the efficacy of bortezomib-based chemotherapy in reducing free-light-chain production [54,55]. This observation is important, as it supports the view that the primary therapeutic determinant of renal recovery is effective chemotherapy, while the incremental benefit of enhanced extracorporeal free-light-chain removal appears limited according to current randomized evidence.

### 6.6. Comparative Summary of Membrane Platforms

The available evidence supports a description of the removal profile of each platform and does not support a ranking of their clinical value. High-cutoff membranes generally achieve the highest per-session free-light-chain clearance but are associated with substantial albumin losses, require prolonged treatment sessions, and have been linked to increased infection risk in some studies; they may also clear certain antimyeloma agents, although the clinical relevance of this effect remains uncertain. Medium-cutoff membranes combine intermediate clearance with lower albumin loss, with a median albumin loss of 2.9 to 7.3 g per session depending on membrane permeability, against 21 g for a 2.1-m^2^ high-cutoff dialyzer in hemodialysis mode. This contrast is drawn across different populations and assays for which no valid head-to-head quantitative comparison can be made and is therefore indicative of order of magnitude alone. These membranes are compatible with standard dialysis platforms, and the myeloma experience covers both isotypes. Lambda reduction over the whole treatment period marginally exceeded that recorded with high-cutoff membranes in the same cohort, though these results were confined to only five high-cutoff patients [65]; that experience nonetheless rested on a small uncontrolled series, and no randomized trial has tested these results. PMMA membranes add an adsorptive mechanism that facilitates effective kappa light-chain removal, with more variable clearance of lambda chains; available data, including the Lyon cohort, derive exclusively from uncontrolled series. HFR-Supra achieved moderate clearance without a requirement for albumin replacement in the series reported, and its evidence base is limited to small observational series; for the HFR-Supra series of 13 patients, only cumulative treatment-cycle reductions were reported, whereas per-session values are available from other HFR configurations and should not be considered interchangeable. None of the newer platforms has been shown to improve dialysis independence, renal function, or survival relative to conventional high-flux hemodialysis, and any statement of a clinical advantage for medium-cutoff, PMMA, or HFR-Supra dialysis would outrun the data; the evidence supports their evaluation in randomized trials. Conventional high-flux hemodialysis therefore remains the standard modality when kidney replacement therapy is otherwise indicated, and it appears sufficient when chemotherapy induces a rapid hematologic response. One structural caveat applies across this comparison. The characterization of these membranes, the pharmacokinetic studies that established high-cutoff performance, and both randomized trials were conducted with the participation, as coauthors, funders or suppliers, of the manufacturer of the dialyzers and of the manufacturer of the free-light-chain assay that defined the outcome. The data are not thereby invalidated, but a substantial proportion of the membrane-characterization literature involves manufacturers or assay developers, highlighting the need for additional independent comparative studies. Table 2 summarizes the comparative characteristics of the membrane platforms discussed above.

**Table 2:** Reported free-light-chain removal and albumin findings for dialysis membrane platforms in myeloma cast nephropathy, with the highest level of clinical-outcome evidence available for each.

Membrane	Dialyzer Studied	Reported Cutoff (kDa) †	Reported κ FLC Reduction	Reported λ FLC Reduction	Albumin Findings	Clinical-Outcome RCT in Cast Nephropathy
High-flux polysulfone	MYRE control arm; Fresenius FX CorDiax 80 (1.8 m^2^)	≈20 (conventional high-flux class) [41]	35% (IQR 19–48), first session [54] ^a^; 36.4% [63] ^b^	18% (IQR 6–56), first session [54] ^a^; 12.9% [63] ^b^	Measured albumin loss in dialysate: ≈0.2 g/session [63] ^b^	Yes (randomized comparator only)
High-cutoff (HCO)	Gambro Theralite (2.1 m^2^); HCO 1100 (1.1 m^2^)	45 (HCO 1100) [41]; 40 post-blood contact (Theralite) [62] ^c^	77% (IQR 67–86), first session [54] ^a^	63% (IQR 52–72), first session [54] ^a^	Measured loss: median 21 g/session (HD), 63 g/session (HDF) [50] ^a^. Reported albumin replacement requirement: 12 g (single dialyzer), 45 g (tandem dialyzers), during 8-h treatment [48] ^a^	Yes (device and prescription differed: MYRE, single Theralite; EuLITE, two HCO 1100 in series)
Medium-cutoff (MCO)	Theranova 400 (1.7 m^2^, clinical); separate MCO prototypes (characterization)	18.1–25 (prototypes, post-blood contact; not Theranova 400) [62] ^d^	44.8 ± 19.5% per session (case series, 3 patients) [64] ^a^; 70% (IQR 39–81) [65] ^a g^; 66.3–70.4% [63] ^b^	37% (IQR 19–50) [65] ^a g^; 42.5–51.5% [63] ^b^	Measured albumin loss in dialysate: 2.9 g/session (Theranova 400) to 7.3 g (most permeable prototype) [63] ^b^	No; case series and retrospective cohorts only
PMMA adsorptive	Toray Filtryzer BK-F (2.1 m^2^)	20 [69] ^b^; 60 [70] ^a e^	55.3 ± 3.4% [69] ^b^	37.8 ± 17.3% [69] ^b^	No albumin replacement required [70] ^a^; serum albumin unchanged [69] ^b^	No; crossover clearance study and retrospective cohorts only
HFR	HFR (Bellco): polyphenylene hemofilter 0.7 m^2^ + styrenic resin cartridge + low-flux filter 1.7 m^2^	≈35 (1st filter) [69] plus resin adsorption	44.1 ± 4.3% [69] ^b^	30.3 ± 2.9% [69] ^b^	Serum albumin unchanged [69]	No; crossover clearance study in patients without myeloma only
HFR-Supra	Synclear 0.2 (0.7 m^2^) + styrenic resin cartridge (Bellco)	≈45–60 (configuration- and definition-dependent) [72,73,74]	≈85% cumulative, involved κ FLC [75] ^f^; 57.6 ± 10% (κ, n = 5) [72] ^a^	≈40% cumulative, involved λ FLC [75] ^f^; 33.5 ± 25% (λ, n = 4) [72] ^a^	No albumin replacement required [75] ^a^; serum albumin unchanged [72]	No; observational series of 13 and 9 patients only

FLC, free light chain; HD, hemodialysis; HDF, hemodiafiltration; PMMA, polymethylmethacrylate; RCT, randomized controlled trial. The table describes reported free-light-chain removal and must not be read as a ranking of clinical effectiveness. Only the high-cutoff row is supported by randomized outcome data, and those trials found no benefit for their primary endpoint. Reduction ratios are not suitable for formal cross-study ranking, because the populations, treatment duration, dialysis prescription, free-light-chain assays, and correction for hemoconcentration differ substantially across the studies cited; this caveat applies with particular force to the comparison between MCO and high-cutoff membranes and between PMMA and HFR configurations. Measures of dispersion are reproduced as reported by the original studies. In small series with repeated dialysis sessions, they do not necessarily represent independent between-patient variability. The unit of analysis was not uniform across studies; in different reports, it was the patient, the dialysis session, or a patient-level average across repeated sessions. † Cutoff values are not comparable across rows. They derive from different methods and conventions, and, where obtained by dextran sieving, they shift by up to an order of magnitude once the membrane is exposed to blood. Dextran sieves differently from globular proteins; so, these figures should not be read against the molecular weight of free light chains. Section 6.1 gives the corresponding hydrodynamic radii, which afford a closer comparison, although those pore radii are themselves dextran-derived and carry the same bias. ^a^ Measured in patients with myeloma-associated acute kidney injury. ^b^ Measured in prevalent hemodialysis patients without monoclonal gammopathy; kinetics at monoclonal free-light-chain concentrations may differ; Donati et al. used a randomized-sequence crossover design and reported per-session reductions rather than clinical outcomes. ^c^ Pristine value 300 ± 100 kDa. ^d^ Range across four MCO prototype membranes after 40 min of blood exposure (pristine values 56–99 kDa); the commercial Theranova dialyzer was not among the membranes characterized. ^e^ The two sources disagree; see Section 6.2. ^f^ Cumulative reduction over the treatment cycle (mean 10 sessions); per-session reduction not separately reported. ^g^ Single-dialysis data reported for a subset of 10 patients; reductions over the whole treatment period in the full cohort are given in Section 6.1. The cutoff reported for Supra-HFR varies with device generation, source, and characterization method; Pendón et al. did not report a nominal cutoff and inferred a value near the molecular weight of albumin from an albumin sieving coefficient of 0.11.

## 7. Timing of Dialysis Initiation and Duration of Extracorporeal Treatment

The decision to initiate dialysis in patients with myeloma and rising creatinine remains guided by conventional indications, including volume overload unresponsive to fluid restriction, refractory hyperkalemia, severe metabolic acidosis, or uremic symptoms [5]. The specific aim of free-light-chain removal has not been shown to justify a lower threshold for dialysis initiation, and the value of dialyzing patients without conventional indications to accelerate light-chain clearance remains undetermined. The MYRE protocol incorporated early initiation of intensive dialysis within the screening period, but its design does not permit evaluation of timing as an independent variable [54].

The kinetics of free-light-chain removal warrant particular attention. In a small Dutch cohort of 15 patients treated with HCO hemodialysis, kappa free-light-chain clearance declined substantially during individual sessions, probably reflecting membrane fouling [78]. In this series, renal recovery occurred in 11 of 15 patients (73%). Among the four patients without recovery, three had persistently elevated free-light-chain concentrations (>5 g/L) beyond two weeks after dialysis initiation, and first-session reductions were not significantly different from those observed in patients who ultimately recovered renal function [78]. The authors interpreted this pattern as indicative of insufficient hematologic response rather than inadequate dialytic clearance, and they suggested consideration of intensifying anti-myeloma therapy when free-light-chain concentrations remain persistently elevated, an interpretation consistent with the IMWG recommendations [5,78].

More broadly, these observations support the principle that extracorporeal removal of free light chains is clinically meaningful only when the underlying plasma cell clone is effectively suppressed. Therefore, chemotherapy should be initiated before or concurrent with dialysis.

Treatment protocols vary across studies. MYRE used eight 5 h sessions over 10 days followed by thrice-weekly treatment; EuLITE used prolonged HCO sessions (6–8 h); and the Lyon PMMA cohort employed six 6 h sessions per week for up to three weeks [54,55,70]. No head-to-head comparison of these schedules has been performed.

An important but often under-discussed practical consideration is the timing of drug administration relative to dialysis sessions. Antimyeloma agents such as bortezomib and dexamethasone may be administered after high-cutoff or medium-cutoff dialysis sessions when feasible, to minimize potential drug removal, although this approach is based on limited pharmacokinetic data [53].

## 8. Renal Prognosis and Its Determinants

Recovery of renal function in cast nephropathy is closely associated with the hematologic response. The studies summarized in this section describe that association in cohorts in which free-light-chain reduction was mainly the product of chemotherapy; they do not test the causal contribution of extracorporeal removal, and they are not evidence that enhancing removal will improve renal recovery. Hutchison and colleagues demonstrated that the magnitude of sFLC reduction was strongly associated with renal recovery; in 39 patients with biopsy-proven myeloma kidney, a 60% reduction by day 21 was associated with a renal recovery rate of approximately 80%, and at day 21, the chances of renal recovery for patients with a reduction greater than 50% were approximately thirteen-fold higher than for those with lesser reductions (with a corresponding sevenfold increase at day 12) [16].

The speed of this reduction is also clinically relevant. The Nordic Myeloma Study Group reported that patients whose renal function recovered had survival approaching that of patients without renal impairment, whereas persistent dialysis dependence beyond three months was associated with markedly worse outcomes [6]. In the Lyon PMMA cohort, a reduction in free light chains exceeding 50% by day 21 was a strong predictor of both renal recovery and survival [70].

Kidney biopsy data provide additional prognostic granularity. Royal et al., in a multicenter retrospective study of 178 patients with light-chain cast nephropathy, reported that both the degree of interstitial fibrosis and tubular atrophy and the density of cortical casts per square millimeter independently predicted renal outcome, together with age, β2-microglobulin, and best hematologic response [13]. This suggests that both acute cast burden and chronic tubulointerstitial damage determine the ceiling for functional recovery. A single-center Vienna cohort of 28 patients with biopsy-confirmed cast nephropathy found that renal responders were younger (median age 61 vs. 77 yr), had higher overall survival (153 vs. 58 months), and were more likely to achieve at least a partial hematologic response [79]. Dialysis dependence at presentation did not preclude recovery, but persistence beyond three months was predominantly observed in non-responders [79].

A recent retrospective analysis from the Princess Margaret Cancer Centre in Toronto provides a contemporary benchmark. Among 70 newly diagnosed patients with dialysis-requiring renal impairment treated between 2010 and 2022 with novel agent-based induction (bortezomib-based in 96%), 33 achieved dialysis independence and 51 proceeded to autologous stem cell transplantation [80]. Early attainment of very good partial response, within 4.4 months, was the sole univariate predictor of dialysis independence (HR 4.17; *p* = 0.0014). At a median follow-up of 39 months, the estimated median overall survival was 98.3 months, with 2- and 5-yr overall survival rates of 84% and 67%, respectively [80]. On multivariable analysis, autologous stem cell transplantation (HR 0.21; *p* = 0.0016), dialysis independence (HR 0.31; *p* = 0.0112), and lower lactate dehydrogenase at diagnosis (*p* = 0.0092) independently predicted survival.

Registry data support a gradual improvement in outcomes over time. Decourt et al. analyzed 1459 patients registered in the French Renal Epidemiology and Information Network (REIN) registry between 2002 and 2011 with end-stage kidney disease attributed to cast nephropathy, light-chain deposition disease, or light-chain amyloidosis. Renal recovery occurred in 133 (9.1%) after a median of five months on dialysis and was more frequent in cast nephropathy (11.3%) than in light-chain deposition disease (8.4%) or amyloidosis (3%). Survival remained poor throughout but improved across the study period, and renal recovery increased after 2006 [81]. Laforet et al. similarly showed progressive increases in dialysis independence rates with the introduction of bortezomib, thalidomide, and lenalidomide [82].

## 9. Open Questions and Future Directions

The evidence in this field remains difficult to reconcile. The pathophysiological logic of extracorporeal free-light-chain removal is biologically plausible, because free light chains are the effector molecules of cast nephropathy and reducing their tubular concentration should, in principle, prevent or reverse cast formation. Yet neither of the completed randomized trials demonstrated a benefit for the primary endpoint, and the safety signal in EuLITE raises concerns about potential net harm in some settings.

Several explanations have been advanced. The trials may have been underpowered for a genuine but modest treatment effect, a plausible concern given that MYRE enrolled 98 patients and EuLITE 90 and that both studies had wide confidence intervals around their primary endpoints. The chemotherapy regimen in both trials may have been sufficiently effective that any incremental contribution of enhanced light-chain removal was difficult to detect, a hypothesis that would predict a larger benefit in patients with chemo resistant disease, a population that was not specifically addressed in these trials. The adverse effects of high-cutoff membranes, including albumin depletion, calcium and magnesium wasting, immunoglobulin loss, and potential drug removal, may have attenuated any potential renal benefit, although the contribution of immunoglobulin loss to infection risk has not been established in these trials [56,57]. Medium-cutoff membranes may sidestep some of these limitations, and their evaluation in a properly designed randomized trial is warranted.

The advent of daratumumab-based regimens may alter this calculus. In a recent series every patient reached a free-light-chain reduction exceeding 50% at a median of three days, whether treated with a daratumumab–bortezomib–cyclophosphamide–dexamethasone quadruplet or a daratumumab–bortezomib–dexamethasone triplet [39]. If chemotherapy alone can achieve light-chain reduction at speeds previously attainable only with chemotherapy plus high-cutoff dialysis, the incremental benefit of extracorporeal removal may diminish further. If a residual role for enhanced dialysis membranes persists, it may be confined to a subset of patients, including those who are anuric or oliguric at presentation, those with a particularly high circulating free-light-chain burden in whom post-dialysis rebound from the extravascular pool is substantial [41,78], those with chemoresistant clones, and patients in whom daratumumab-based regimens are contraindicated or unavailable. Each of these subsets respresents a hypothesis derived from pharmacokinetic reasoning and uncontrolled series, none has been tested prospectively, and no threshold of free-light-chain concentration or kinetics has been validated to define any of them.

The populations across which this literature is spread are also not interchangeable, and the distinction bears directly on the extrapolation of the high-cutoff trials to current practice. MYRE and EuLITE enrolled newly diagnosed patients with biopsy-proven cast nephropathy, treated with bortezomib-based regimens before anti-CD38 antibodies were available, and their results describe that setting alone [54,55]. DARE and GMMG-DANTE recruited relapsed or refractory disease selected on renal impairment [37,38]; the frontline series of Kim et al. and of Horigome et al. included newly diagnosed patients with clinically presumed cast nephropathy and no requirement of biopsy confirmation [39,40]; biopsy status is reported inconsistently across the newer-membrane cohorts [64,65,70,75]; and renal-response definitions differ across all of these. The speed of sFLC reduction with anti-CD38-containing induction would predict a smaller incremental value of extracorporeal removal in the contemporary era than in the bortezomib era, but none of these datasets can test this, and analyzing this issue will require a randomized comparison conducted with a contemporary chemotherapy backbone.

Ongoing prospective work will further refine this picture. The ECOG-ACRIN EAA241 study (NCT07085728), a randomized phase 2 trial comparing daratumumab-hyaluronidase–bortezomib–dexamethasone against usual-care cyclophosphamide–bortezomib–dexamethasone in newly diagnosed MM with biopsy-proven or clinically presumed light-chain cast nephropathy and new-onset renal failure, represents the first randomized effort to test whether upfront incorporation of an anti-CD38 antibody alters renal recovery in this population [83]. Complementary retrospective evidence is also accumulating. A single-center Columbia University series of 274 consecutively diagnosed MM patients identified 46 (16.8%) with cast nephropathy treated in the modern era, of whom 96% received bortezomib and 67% an anti-CD38 monoclonal antibody as part of frontline therapy [84]. The renal overall response rate was 76.1% (35/46) and the renal complete response rate 32.6% (15/46) at 6 months, and 6-month overall survival in the cast nephropathy cohort was 100%, appearing comparable to that observed in myeloma patients without cast nephropathy within this cohort. These figures derive from an uncontrolled series whose population and renal-response definition differ from those of MYRE and EuLITE, so they cannot be set directly against the control arms of those trials; they are compatible with, but do not establish, a narrowing of the historical survival gap between cast nephropathy and other newly diagnosed myeloma presentations since the integration of anti-CD38 therapy into frontline regimens. These datasets are not yet of sufficient size or design to alter clinical practice, but they make it increasingly plausible that the most consequential advances in renal outcomes will derive from improvements in antimyeloma therapy rather than from extracorporeal membrane choice. Several interventional studies registered on ClinicalTrials.gov reflect this dual research direction. The DREAMM-12 study is evaluating the pharmacokinetics, safety, and tolerability of belantamab mafodotin in patients with relapsed or refractory MM across the full spectrum of renal function, including end-stage renal disease on dialysis, a population systematically excluded from earlier registration trials [85]. Within the extracorporeal arm of the field, the trial NCT05429515 is examining HFR-Supra in MM patients, and additional studies are revisiting plasma exchange and combined approaches with modern antimyeloma regimens [86]. These efforts indicate that clinical equipoise around the role of light-chain removal persists despite the negative primary endpoints of completed randomized trials, and that future research is likely to integrate, rather than oppose, antimyeloma therapy and extracorporeal strategies.

Renal transplantation for patients who remain dialysis-dependent after myeloma remission is an area of growing interest, particularly as life expectancy with novel agents continues to improve. The IMWG update acknowledges this possibility, noting that transplantation is likely to be increasingly considered in selected candidates [5].

## 10. Conclusions

**Figure 1:**
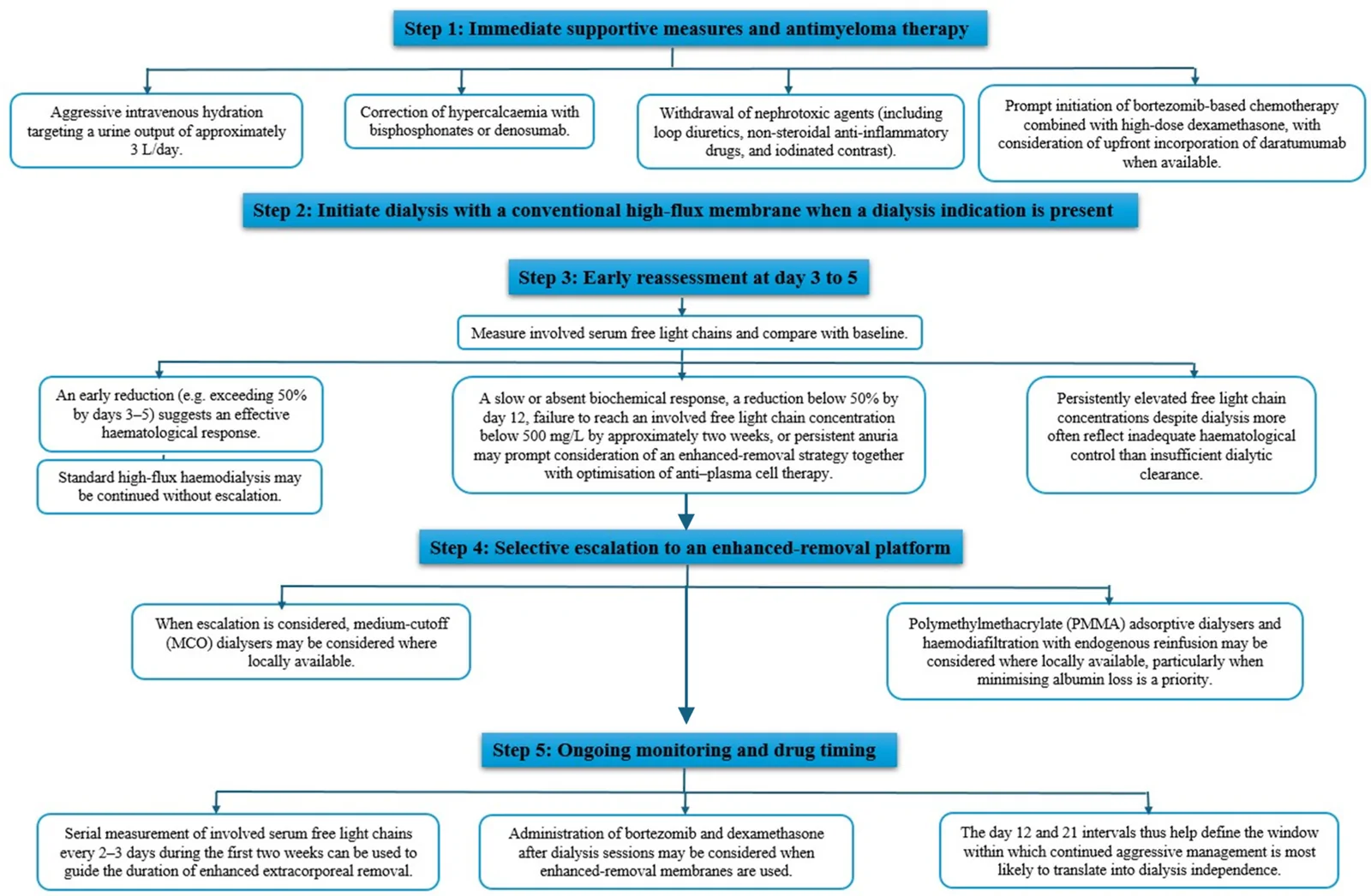
Framework for the management of myeloma cast nephropathy requiring dialysis and for the prospective evaluation of enhanced free-light-chain removal. Steps 1 and 2 reflect consensus recommendations and randomized evidence; steps 3 to 5, including the reassessment at day 3 to 5, describe an expert-opinion, hypothesis-generating framework; its selection criteria and thresholds have not been validated and are proposed for testing in prospective trials, and enhanced free-light-chain removal has not shown clinical superiority over conventional high-flux hemodialysis when effective antimyeloma therapy is started promptly.

From a practical standpoint, myeloma cast nephropathy should be regarded as a renal emergency. Established cornerstones of management include carefully monitored hydration, often targeting approximately 3 L/day in patients without oliguria, congestion, or cardiac contraindications; correction of hypercalcemia with bisphosphonates or, when bisphosphonates are unsuitable because of severe kidney impairment, denosumab, which requires prior correction of vitamin D deficiency and close monitoring for potentially severe hypocalcemia; avoidance of non-steroidal anti-inflammatory drugs and of non-essential iodinated contrast exposure; avoidance of forced diuresis with loop diuretics in hypovolemic patients, while recognizing that these agents remain appropriate when clinically required for volume overload; and prompt initiation of bortezomib-based chemotherapy with high-dose dexamethasone (Figure 1).

Extracorporeal light-chain-removal remains biologically rational, and its clinical value has not been established. The principal evidence-based conclusion of this review is that conventional high-flux hemodialysis, started when a conventional indication for dialysis is present and combined with immediate antimyeloma therapy, is the standard kidney-replacement modality in myeloma cast nephropathy. The two randomized trials found no benefit of enhanced free-light-chain removal for their primary renal endpoint, and their pooled analysis found no benefit for survival. Beyond that conclusion the field is one of unresolved equipoise, in which pathophysiological rationale, pharmacokinetic data, and uncontrolled cohorts suggest that patients with a slow decline in free light chains at early reassessment (3 to 5 days), persistent anuria, or very high pre-dialysis free-light-chain concentrations despite appropriate chemotherapy might be those in whom a time-limited enhanced-removal strategy could still be tested; these criteria are put forward as hypotheses for prospective trials, none of them carries a validated threshold, and they do not constitute a recommendation for current practice. Since daratumumab-containing induction lowered free light chains within days in the small frontline series available, the contemporary question has shifted from the membrane that removes the largest quantity of circulating light chains to the incremental renal benefit, if any, that extracorporeal removal adds to such therapy, and only a randomized trial with a contemporary chemotherapy backbone can answer this. Where an enhanced-removal platform is used within a study, the choice among platforms should weigh clearance against albumin loss, local availability, and tolerance of prolonged sessions in patients who are often frail and elderly [87].

## Figures and Tables

**Table 1:** Randomized controlled trials of extracorporeal free-light-chain removal in myeloma cast nephropathy.

Trial	N	Design	Chemotherapy	Intervention vs. Control	Key Results
Zucchelli et al. 1988 [42]	29	RCT, single-center	Corticosteroids + cytotoxic drug (pre-bortezomib)	TPE + HD on-demand vs. PD	Renal recovery 13/15 vs. 2/14; 1-yr survival 66% vs. 28% (*p* < 0.01)
Johnson et al. 1990 [43]	21 (11 TPE, 10 control)	RCT, single-center (Mayo Clinic)	Forced diuresis + chemotherapy (pre-bortezomib)	TPE added vs. forced diuresis + chemotherapy	Faster decline in serum myeloma protein with TPE; among 5 oliguric dialysis-dependent patients, 3 recovered renal function, all in TPE arm
Clark et al. 2005 [44]	104	RCT, open-label; 14 Canadian centers	Heterogeneous (pre-bortezomib)	TPE (5–7 exchanges of 50 mL/kg) vs. conventional Rx	No difference in composite endpoint (death, HD, or reduced eGFR) at 6 months
MYRE (Bridoux et al. 2017) [54]	98 (94 mITT)	RCT, open-label; 48 French centers; biopsy-proven CN; 4–15 d screening	Bortezomib (1.3 mg/m^2^) + dexamethasone	HCO-HD (Theralite 2.1 m^2^; 8 × 5 h/10 d, then 3×/wk) vs. HF-HD	3 mo: 41.3% vs. 33.3% (*p* = 0.42, NS); 6 mo: 56.5% vs. 35.4% (*p* = 0.04); 12 mo: 60.9% vs. 37.5% (*p* = 0.02); OS HR 0.76 (*p* = 0.46)
EuLITE (Hutchison et al. 2019) [55]	90	Phase 2 RCT, open-label; 16 UK/German centers; biopsy-proven CN; no screening phase	Bortezomib (1 mg/m^2^) + doxorubicin + dexamethasone	HCO-HD (2 × HCO-1100 in series, 2.2 m^2^; 6 h first, then 8 h, 7 sessions in first 10 d) vs. HF-HD	90 d: 56% vs. 51% (RR 1.09; *p* = 0.81); 12-mo myeloma response 42% vs. 68% (*p* = 0.022); lung infection RR 4.64 (post hoc; *p* = 0.008); age-adjusted OS HR 2.63 (1.13–6.15; *p* = 0.03)

HD, hemodialysis; HCO, high-cutoff; HF, high-flux; CN, cast nephropathy; TPE, therapeutic plasma exchange; PD, peritoneal dialysis.

## Data Availability

No new data were created or analyzed in this study. Data sharing is not applicable to this article.

## Abbreviations

AKI, acute kidney injury; CDR3, complementarity-determining region 3; CI, confidence interval; CN, cast nephropathy; CRP, C-reactive protein; eGFR, estimated glomerular filtration rate; EuLITE, European Trial of Free Light Chain Removal by Extended Haemodialysis in Cast Nephropathy; FLC, free light chain; GFR, glomerular filtration rate; HCO, high-cutoff; HD, hemodialysis; HDF, hemodiafiltration; HF, high-flux; HFR, hemodiafiltration with endogenous reinfusion; HR, hazard ratio; IL-6, interleukin-6; IMWG, International Myeloma Working Group; IQR, interquartile range; MCO, medium-cutoff; MCP-1, monocyte chemoattractant protein-1; mITT, modified intention-to-treat; MM, multiple myeloma; NF-κB, nuclear factor-κB; NSAID, non-steroidal anti-inflammatory drug; OR, odds ratio; OS, overall survival; PD, peritoneal dialysis; PE, plasma exchange; PEPA, polyester polymer alloy; PMMA, polymethylmethacrylate; RCT, randomized controlled trial; REIN, Renal Epidemiology and Information Network; RR, relative risk; RRMM, relapsed or refractory multiple myeloma; sFLC, serum free light chain; TGF-β, transforming growth factor-beta; TPE, therapeutic plasma exchange; VAD, vincristine–adriamycin–dexamethasone; VCd, bortezomib–cyclophosphamide–dexamethasone; Vd, bortezomib–dexamethasone.
